# A reproducible simulation dataset and benchmark for parametric competing-risks inference under censoring and latent dependence

**DOI:** 10.1016/j.dib.2026.113340

**Published:** 2026-10-09

**Authors:** Sabrigiriraj M, Manoharan K, Senthil Madasamy N, Govindaraj V, Prathipa K, Ravi T

**Affiliations:** a Department of Information Technology, Hindusthan College of Engineering and Technology, Valley Campus, Pollachi Highway, Othakkalmandapam, Coimbatore, Tamil Nadu, 641032, India; b Department of Electronics and Communication Engineering, SNS College of Technology, SNS Kalvi Nagar, Sathy Main Road, Vazhiyampalayam, Saravanampatti Post, Coimbatore, Tamil Nadu, 641035, India; c Department of Computer Science and Engineering, Dr. Mahalingam College of Engineering and Technology, Mackkinaickenpatti Post, Udumalai Road, Pollachi, Coimbatore, Tamil Nadu, 642003, India; d Department of Electronics and Communication Engineering, Kalaignarkarunanidhi Institute of Technology, Kannampalayam Post, Coimbatore, Tamil Nadu, 641402, India; e Department of Computer Science and Engineering, Shree Venkateshwara Hi-Tech Engineering College, Erode-Gobi Main Road, Sri Kalivani Nagar, Othakuthirai, Gobichettipalayam, Erode, Tamil Nadu, 638455, India; f Department of Computer Science and Engineering, Vel Tech Rangarajan Dr. Sagunthala R&D Institute of Science and Technology, No. 42, Avadi-Vel Tech Road, Vel Nagar, Avadi, Chennai, Tamil Nadu, 600062, India

**Keywords:** Wald test, Likelihood-ratio test, Score test, Survival analysis, Copulas, Boundary estimates, Monte Carlo uncertainty

## Abstract

This article describes a simulation dataset for evaluating parametric competing-risks inference under censoring and latent dependence. The dataset was generated in Python and contains 648,000 replicate records from 303 block-specific scenarios, representing 291 unique statistical conditions. The design includes exponential, Weibull and Gompertz lifetime models, two, three and five competing causes, and sample sizes ranging from 20 to 200. Type-I, Type-II and hybrid censoring are considered. A separate dependence block uses Clayton, Frank and Gumbel copulas with Kendall’s tau values of 0.25, 0.50 and 0.75, with inference performed under a working-independence assumption. Replicate records contain estimates, test statistics and availability indicators for Wald, likelihood-ratio and score tests. The release provides 303 Parquet partitions, scenario and parameter registries, summaries of bias and root mean squared error, Monte Carlo uncertainty estimates, and bootstrap coverage checks. A representative subject-level sample contains 35,250 rows. Supporting materials include an executed notebook, figures, a field-level data dictionary, validation records and file checksums. Recorded identifiers and deterministic seeds support regeneration of individual simulated studies. Researchers can use the data to evaluate statistical procedures across the documented conditions, examine test availability and boundary cases, reproduce benchmark calculations, and develop teaching examples.

Specifications Table

**Table:** 

Subject	Computer Sciences
Specific subject area	Parametric competing risks, censoring and finite-sample inference.
Type of data	Synthetic subject-level and replicate-level tables; analysed summaries, figures and code. Formats: CSV, Parquet, JSON, PNG, Markdown, plain text and Jupyter/Google Colab notebook (.ipynb) .
Data collection	Deterministic Monte Carlo simulation in Python 3.13 generated latent failure times under specified lifetime models, copulas and censoring rules. Scenario-specific seeds were used to generate replicate records, calculate estimates and test statistics, and produce validation summaries. The executed Colab notebook records the software environment.
Data source location	Department of Information Technology, Hindusthan College of Engineering and Technology, Coimbatore, Tamil Nadu, India. All observations were generated synthetically*.*
Data accessibility	Repository name: Mendeley Data Data identification number: 10.17632/yd2x2wkgdk.1 Direct URL to data: https://data.mendeley.com/datasets/yd2x2wkgdk/1 Instructions for accessing these data: Open the repository record and download Version 1. Data are available under CC BY 4.0; code is provided under the MIT licence.The dataset is publicly available under the CC BY 4.0 licence
Related research article	None*.*

## 1 Value of the Data

• The benchmark gives replicate-level results for Wald, likelihood-ratio and score tests under one documented design.
• It makes small-sample error, censoring effects, dependence misspecification and boundary behaviour available for direct study.
• The replicate partitions support evaluation of procedures based on the released counts, exposures, estimates and test statistics. Complete subject-level studies can be regenerated from their recorded seeds when a method needs individual observations.
• Stable scenario identifiers, deterministic replicate seeds, validation records, checksums and the executed notebook support audit and exact regeneration.
• The release records unavailable Wald tests separately from computable likelihood-ratio and score tests, with explicit indicators for zero-event cases.

## 2 Background

In a competing-risks experiment, several causes can end an observed lifetime, but only the first event and its cause are recorded. Such data arise in reliability engineering and survival analysis. Censoring restricts observation and can leave some causes with few recorded events.

Type-I censoring ends observation at a fixed time, whereas Type-II censoring ends it after a specified number of failures. Hybrid schemes combine time and failure-count limits [[1], [2], [3]]. Methods for analysing competing-risks data under these schemes have been examined in earlier work [[4], [5], [6]].

An earlier data article and repository provide simulation data for Wald, likelihood-ratio and score tests in parametric competing-risks models [7,8]. The present dataset was generated independently to provide replicate-level records across a broader set of documented conditions. No records were copied from the earlier release.

The design includes additional copula families, specified Kendall’s tau levels, separate censoring comparisons, five-cause settings and smaller sample sizes. Replicate identifiers, seeds, test-availability indicators and validation files are retained to support reproducibility and inspection of individual simulated studies. The dataset was compiled to support method evaluation and reuse, following established guidance for reporting simulation studies [9].

## 3 Data Description

The dataset and accompanying code are publicly available in Mendeley Data [10].

The release contains 343 files: 341 payload files and two integrity files. Table 1 describes every file outside the scenario-partition folder individually and the 303 identically structured Parquet partitions collectively. The repository has six files at its root, three files in code/, 21 files directly in data/, 303 files in data/replicate_partitions/, eight files in figures/ and two files in manifests/. The internal manifest records the relative path, size and SHA-256 hash of each payload file. The files manifests/file_manifest.csv and manifests/sha256_checksums.txt are excluded from their own inventory.

**Table 1:** Complete inventory of release files and the shared scenario-partition collection.

File or folder	Purpose
README.md	Release overview, version information, file guide, reproduction steps, validation status and reuse guidance.
DATA_DICTIONARY.md	Field definitions, stored types, units, allowed values and missing-value conventions for 27 file schemas, including the shared partition schema.
CITATION.cff	Machine-readable citation metadata for the dataset.
LICENSE_DATA.txt	CC BY 4.0 licence terms for the data, documentation, figure-source values and figures.
LICENSE_CODE.txt	MIT licence terms for the notebook and software code.
environment.json	Recorded software-environment metadata for reproducibility.
code/CRM_competing_risks_REFINED_v2_publication_colab.ipynb	Executed Google Colab/Jupyter notebook for generation, estimation, validation, figure preparation and release packaging.
code/requirements_lock.txt	Pinned software-package requirements for reproducing the computing environment.
code/run_configuration.json	Frozen publication-run configuration and simulation settings.
data/bootstrap_summary.csv	Coverage counts, proportions and Wilson intervals by lifetime model, with the number of valid parent intervals.
data/bootstrap_validation.csv	Parent-specific bootstrap records, requested and successful resample counts, interval endpoints and coverage indicators.
data/boundary_nuisance_cases.csv	Detailed records classified as interior-target nuisance-boundary cases; the release contains 17,127 such records.
data/condition_registry.csv	Mapping of 291 unique statistical conditions to their scenario identifiers, blocks and numbers of scenario records.
data/design_overlap_report.csv	The 12 deliberately repeated reference conditions, identifying the paired scenario records and their blocks.
data/empirical_sample_size.csv	Smallest tested sample sizes meeting point-estimate and Monte Carlo-conservative power targets, with maximum tested sizes and target-reached indicators.
data/generation_current_run.csv	Generation or verified-reuse status and timings from the saved publication execution, including RESUME_VALIDATION records.
data/generation_runtime_report.csv	Preserved original generation status and non-zero generation timings for each scenario.
data/monte_carlo_uncertainty.csv	Test-specific available denominators, rejection counts, Monte Carlo errors, Jeffreys summaries, Wilson intervals and failure bounds.
data/parameter_registry.csv	Cause-specific null and data-generating parameter values for the scenario designs.
data/scenario_registry.csv	Complete design definitions and identifiers for the 303 block-specific scenarios.
data/subject_level_sample.parquet	35,250 representative subject-level rows containing latent times, observed follow-up, event causes and censoring information.
data/subject_level_selection.csv	The 93 selected moderate-censoring null scenarios used for the subject-level sample, with their identifiers and design settings.
data/summary_bias_rmse.csv	Cause-specific estimation bias and root mean squared error, summarized by scenario.
data/summary_size_power.csv	Scenario-level test availability, rejection proportions, interval coverage, boundary frequencies and design summaries.
data/t0_calibration.csv	Null-model calibration records for the Type-I time limits used in Type-I and hybrid censoring.
data/target_boundary_cases.csv	Detailed records with the cause-1 estimate on the boundary; the release contains 361 such records.
data/technical_validation_summary.csv	Scenario counts, rejection-proportion ranges and maximum boundary rates grouped by simulation block and alternative type.
data/test_validity_issues.csv	Replicate identifiers, seeds, status and test-availability flags for records with at least one unavailable test.
data/theoretical_reference.csv	Empirical rejection proportions and information-based asymptotic reference calculations, including differences, uncertainty measures and comparison flags.
data/validation_report.csv	108 programmed validation records covering the generation, dependence and reproducibility checks.
data/replicate_partitions/	303 Parquet files, one per scenario, named SCN_<16-character hexadecimal identifier>.parquet; together they contain 648,000 replicate records with the common schema described below.
figures/figure_1_power.png	Fig. 1: fixed-alternative rejection proportions under moderate hybrid censoring.
figures/figure_1_power_source.csv	Source values for Fig. 1: scenario identifiers, model, sample size, test-specific denominators, rejection proportions and Monte Carlo errors.
figures/figure_2_type1_error.png	Fig. 2: null rejection proportions in the correctly specified independent core.
figures/figure_2_type1_error_source.csv	Source values for Fig. 2: scenario identifiers, model, censoring profile, sample size, denominators and rejection proportions.
figures/figure_3_dependence.png	Fig. 3: null rejection proportions under working-independence misspecification.
figures/figure_3_dependence_source.csv	Source values for Fig. 3: scenario identifiers, model, copula, Kendall’s tau, sample size, denominators and rejection proportions.
figures/figure_4_boundary_rates.png	Fig. 4: target-boundary and interior-target nuisance-boundary classification rates.
figures/figure_4_boundary_source.csv	Source values for Fig. 4: scenario identifiers, model, cause count, sample size and target-boundary, nuisance-boundary and fully regular rates.
manifests/file_manifest.csv	Individual release-relative paths, byte sizes and SHA-256 hashes for the 341 payload files; excludes both integrity files.
manifests/sha256_checksums.txt	SHA-256 verification entries for the same 341 payload files; excludes both integrity files.

DATA_DICTIONARY.md is generated from the completed release. It documents every field in the CSV products, the representative subject-level Parquet file, the common replicate-partition schema, the figure-source tables and the manifest. It also explains structural placeholders and test-specific missing values.

All 303 scenario partitions use the naming pattern data/replicate_partitions/SCN_<16-character hexadecimal identifier>.parquet. The filename stem is the scenario_id; for example, SCN_00c603827b11969b.parquet corresponds to scenario_id SCN_00c603827b11969b. The identifier links each partition to data/scenario_registry.csv. The condition_id field links statistically identical conditions across blocks through data/condition_registry.csv, while data/design_overlap_report.csv identifies the deliberately repeated reference pairs. The manifest lists every individual partition filename and its checksum.

Each partition has 51 fields covering pipeline and scenario identifiers, replicate identifiers and seeds, execution status, stopping rules, observed failure counts and fractions, estimate and test availability, cause-specific event counts, cumulative exposures and coefficient estimates, log likelihoods, Wald, likelihood-ratio and score statistics, p-values, rejection indicators, information terms, standard errors, interval limits and coverage. The common schema is documented once in DATA_DICTIONARY.md and applies to all partitions. Cause columns above the scenario-specific number of causes are structural placeholders, not observed zero-event causes. Null scenarios contain 3000 replicates and alternative scenarios contain 1500 replicates.

The subject-level sample is linked to its selected scenarios through data/subject_level_selection.csv. The four PNG files in figures/ correspond to Fig. 1, Fig. 2, Fig. 3, Fig. 4 in this article; each has the separately named source CSV listed in Table 1. The source files retain scenario identifiers and the values used for plotting. The code/ folder contains the executed notebook, frozen settings and package requirements; environment.json records the software environment. README.md, CITATION.cff and the two licence files document reuse and attribution.

**Fig. 1:**
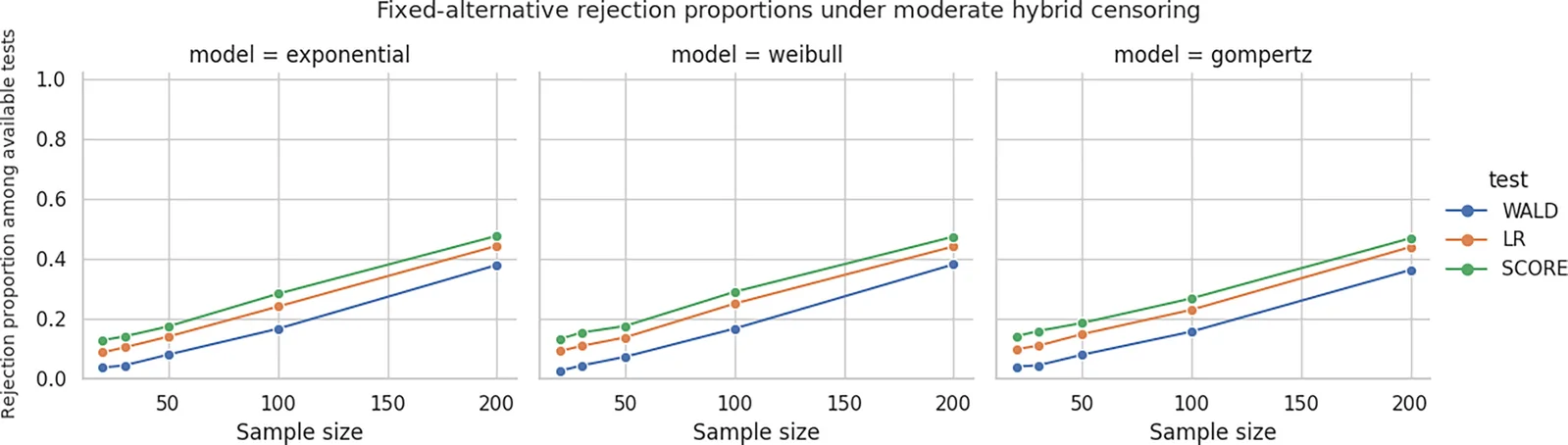
Fixed-alternative rejection proportions under moderate hybrid censoring. The first coefficient is increased by 25%. Each test uses its own available denominator.

**Fig. 2:**
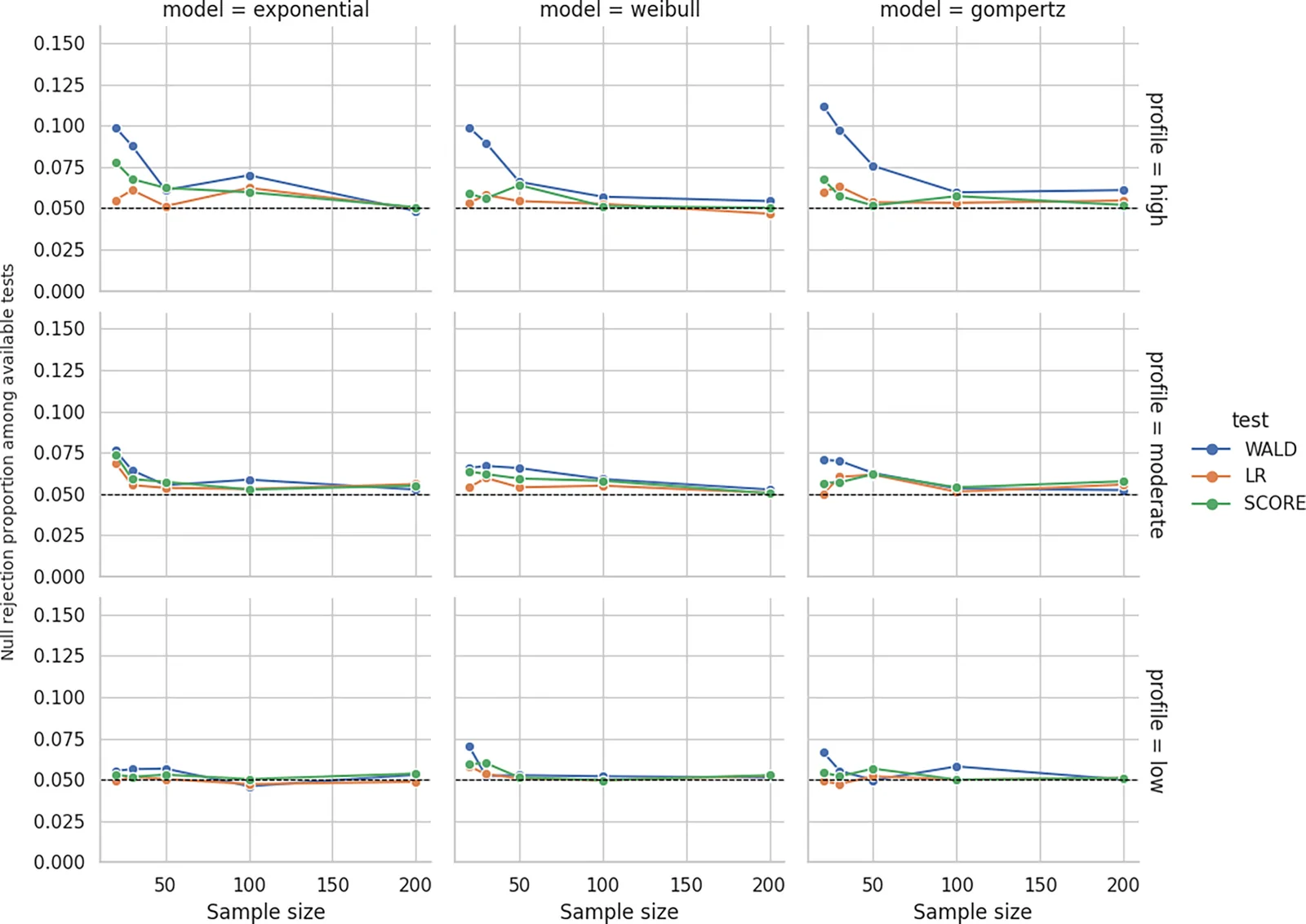
Null rejection proportions in the correctly specified core block. The dashed line marks 0.05. Panels distinguish lifetime models and censoring profiles. Each test uses its own available denominator.

**Fig. 3:**
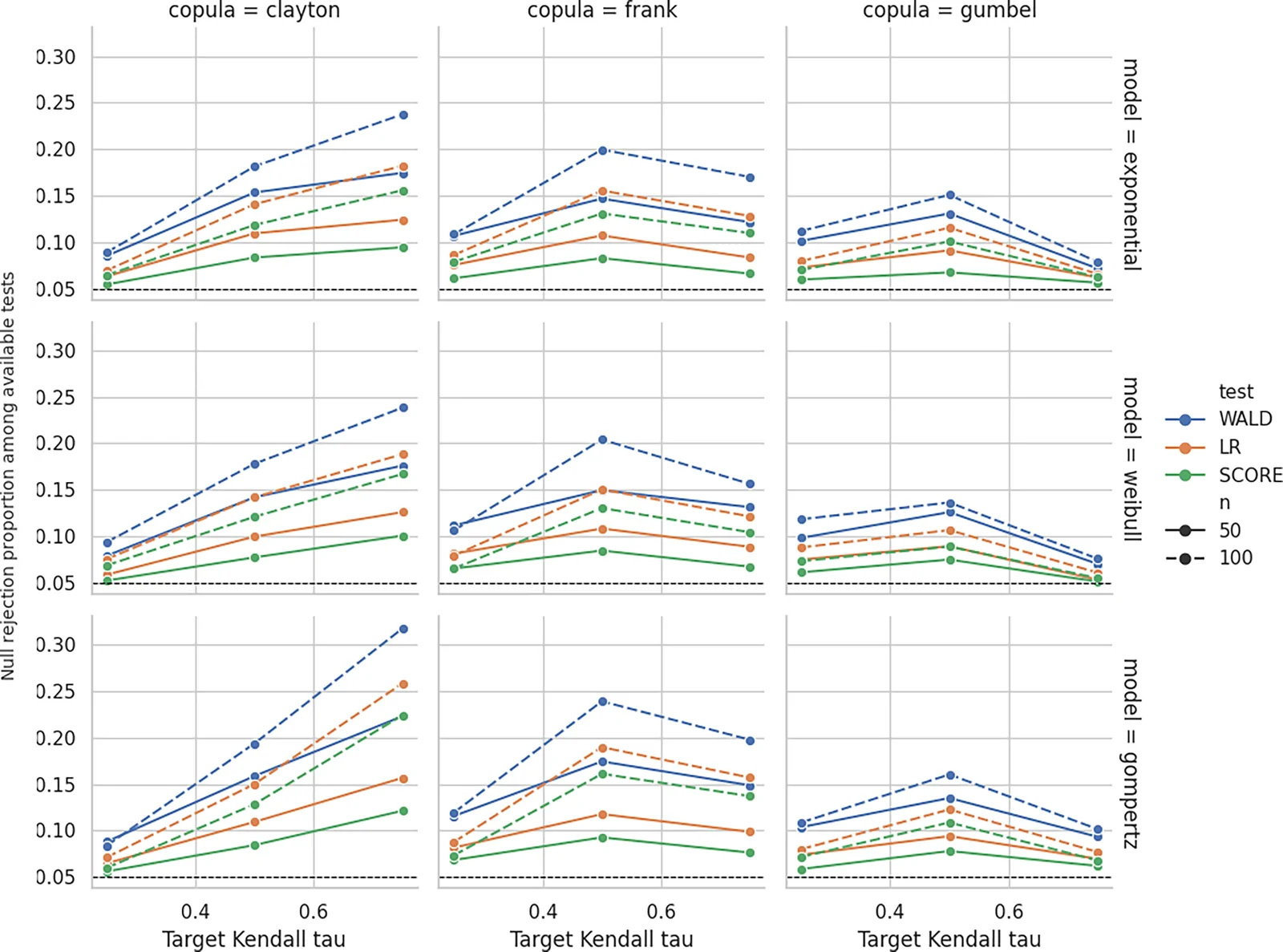
Null rejection proportions when dependent latent times are analysed with a working-independence likelihood. Solid lines represent n = 50 and dashed lines represent n = 100. Each test uses its own available denominator.

**Fig. 4:**
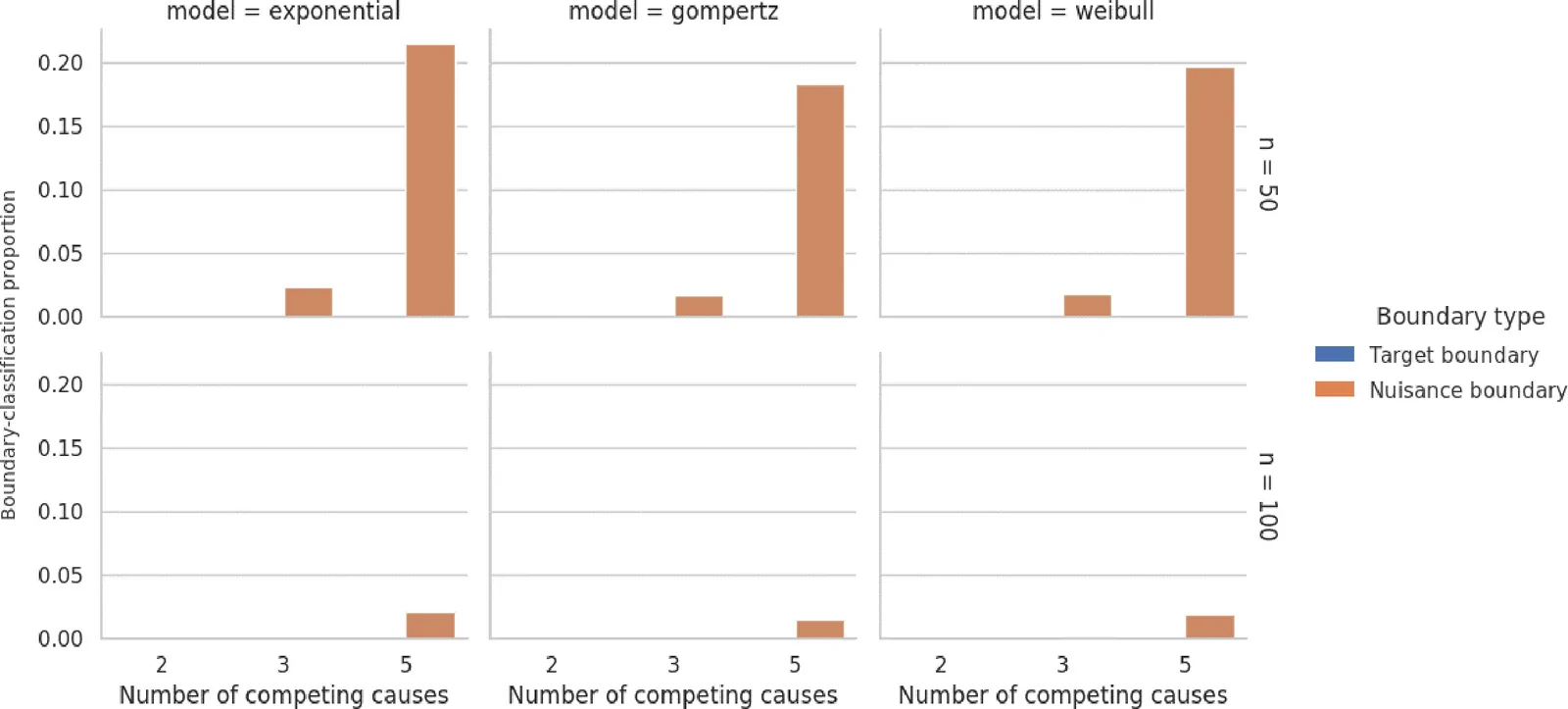
Target-boundary and interior-target nuisance-boundary classification rates in independent moderate hybrid null conditions, grouped by lifetime model, number of competing causes and sample size.

### 3.1 Comparison with the earlier dataset

Table 2 compares the structure and scenario coverage of the earlier dataset [7,8] with the present benchmark.

**Table 2:** Comparison of the earlier dataset and the present benchmark.

Feature	Earlier dataset	Present benchmark
Released structure	Mainly analysis tables	Replicate partitions, summaries and subject sample
Scenario identity	Original design files	303 hashed scenario records; 291 unique conditions
Lifetime models	Exponential, Weibull, Gompertz	Exponential, Weibull, Gompertz
Sample sizes	50, 100, 200, 500	20, 30, 50, 100, 200 in the core
Competing causes	Two or three	Two, three or five
Dependence	Clayton robustness	Clayton, Frank and Gumbel
Dependence scale	Copula parameters	Kendall tau of 0.25, 0.50 and 0.75
Censoring	Hybrid focus	Separate Type-I, Type-II and hybrid blocks
Uncertainty	Simulation summaries	MCSE, Wilson intervals and failure bounds

Twelve statistical conditions are generated independently in both the core and censoring-comparison blocks. These form 24 scenario records, or 12 paired reference conditions. They are retained on purpose. Agreement between these independent pairs is used as an internal Monte Carlo check.

### 3.2 Validation records

The release-level completion and reproducibility checks are summarized in Table 3. All programmed validation checks passed. The 303 scenario summaries reconciled with their partitions, all 465 subject-level scenario-replicate groups reconciled, and all 27 independent closed-form calculations agreed with the stored values. The earlier validation checks also passed: 27 full-record seed regenerations, 30 marginal-transform checks, nine copula checks and 36 independent reference comparisons. The validation confirmed 361 target-boundary records and 17,127 interior-target nuisance-boundary classifications.

**Table 3:** Release-level completion and reproducibility checks.

Validation item	Observed result	Assessment
Replicate records	648,000	Complete
Scenario partitions	303 of 303	Passed
Target-boundary records	361 (0.056%)	Recorded
Interior-target nuisance-boundary classifications	17,127 (2.64%)	Recorded
Subject-level sample	35,250 rows	Complete
Copula checks	9 of 9	Passed
Full-record seed regeneration	27 of 27	Passed
Independent anchor agreement	36 of 36	Passed
Marginal-transform checks	30 of 30	Passed
Bootstrap parent intervals	90	Complete
Manifest-verified payload files	341	Passed
Scenario-summary reconciliation	303 of 303	Passed
Subject-level group reconciliation	465 of 465	Passed
Independent closed-form calculations	27 of 27	Passed

Bias and RMSE were calculated separately for every cause and scenario and are supplied in data/summary_bias_rmse.csv. The summaries retain the coefficient scale of each lifetime model and link to the scenario registry and Monte Carlo uncertainty records.

### 3.3 Fixed-alternative rejection proportions

The fixed-alternative rejection proportions are shown in Fig. 1. Rejection proportions increased with sample size in all three lifetime models. Under moderate hybrid censoring, the score test usually had the highest rejection proportion, followed by the likelihood-ratio test and then the Wald test. At n = 200, the Wald rejection proportion among available Wald tests was about 0.36–0.38, the likelihood-ratio rejection proportion was about 0.43–0.45, and the score rejection proportion was about 0.46–0.48. None of the test-specific rejection proportions reached 0.80 for the 25% increase within the tested range.

### 3.4 Local-alternative records

The 45-core local-alternative scenarios use the absolute shift θ₁(true) = θ₁(null) + 1/√n. Across all models, sample sizes and censoring profiles, the observed rejection proportions ranged from 0.025 to 0.296 for Wald, from 0.063 to 0.351 for likelihood ratio, and from 0.069 to 0.375 for score. The ranges cover conditions with different coefficient scales. Complete scenario-level values and Monte Carlo intervals are provided in data/summary_size_power.csv and data/monte_carlo_uncertainty.csv.

### 3.5 Null rejection under independent sampling

The null rejection proportions in the correctly specified core block are shown in Fig. 2. In the correctly specified core block, null rejection proportions generally moved toward 0.05 as sample size increased. The largest observed Wald null rejection proportion among available Wald tests was 0.112. The largest observed likelihood-ratio null rejection proportion was 0.069, and the largest observed score null rejection proportion was 0.078. The largest departures occurred with small samples and heavy censoring.

Across null scenarios in the independent censoring-comparison block, the Wald rejection proportion among available tests ranged from 0.051 to 0.067, the likelihood-ratio rejection proportion ranged from 0.044 to 0.060, and the score rejection proportion ranged from 0.045 to 0.064.

### 3.6 Working-independence records

Fig. 3 summarizes the null rejection proportions under working-independence misspecification. Under the latent-parameter null, the maximum proportions were 0.317 for Wald, 0.258 for likelihood ratio and 0.224 for score. The figure separates Clayton, Frank and Gumbel copulas, Kendall’s tau values and sample sizes.

### 3.7 Boundary classifications

The target- and nuisance-boundary classification rates are shown in Fig. 4. A total of 361 of 648,000 records placed the target coefficient on the boundary. This is 0.056% of the complete release. A total of 17,127 records, or 2.64%, were classified as SUCCESS_WITH_BOUNDARY_NUISANCE. This classification requires an interior cause-1 estimate and at least one zero-event nuisance cause. It is therefore not a count of every nuisance-boundary flag among records already classified as target-boundary cases. In the independent higher-K block, the interior-target nuisance-boundary classification rate was about 18–21% for K = 5 at *n* = 50. It fell to about 1–2% at *n* = 100.

### 3.8 Bootstrap coverage records

Bootstrap percentile-interval coverage for the three lifetime models is recorded in Table 4. Of 30 parent intervals per model, 29 covered the true coefficient for the exponential model, 27 for Gompertz and 26 for Weibull. The table reports the corresponding proportions and Wilson 95% intervals.

**Table 4:** Bootstrap percentile-interval coverage by lifetime model.

Model	Covered / 30	Coverage	Wilson 95% interval
Exponential	29	0.967	0.833–0.994
Gompertz	27	0.900	0.744–0.965
Weibull	26	0.867	0.703–0.947

### 3.9 Access and reuse

The dataset is publicly available in Mendeley Data under the Creative Commons Attribution 4.0 International (CC BY 4.0) licence (DOI: 10.17632/yd2x2wkgdk.1). The generation code is provided under the MIT licence. The SHA-256 checksum of the deposited release ZIP is 42f07751c4143517503bc64975f424c938a7b9fc153544f63808bba2eceb4f40. This archive-level checksum verifies the ZIP file exactly as deposited. After extraction, the 341 payload files can be verified using manifests/file_manifest.csv and manifests/sha256_checksums.txt; these two manifest files are intentionally excluded from their own inventory.

The replicate partitions contain counts, exposures, estimates and test statistics. Methods requiring complete individual observations can use the representative subject-level file for initial work or regenerate studies from the recorded seeds. The partitions alone do not contain the full subject-level observations for every replicate.

Begin with data/scenario_registry.csv. Filter by block, model, sample size, censoring profile, copula or alternative.

Use scenario_id to locate the matching Parquet partition. Retain scenario_id and condition_id after combining files.

Use replicate_id and seed to regenerate an individual synthetic study.

Use each test-specific availability flag and denominator. Do not apply the Wald denominator to likelihood-ratio or score results.

Report target-boundary cases and interior-target nuisance-boundary classifications separately.

Treat dependence scenarios as robustness experiments under a misspecified working-independence likelihood.

Use the Wilson intervals and failure bounds when comparing rejection proportions.

Verify the release with manifests/sha256_checksums.txt before analysis.

For a complete fresh run, direct the notebook to a new empty output directory and execute all cells in their original order without changing the master seed or simulation settings. When the output directory contains no valid partitions, the workflow generates all 303 partitions before producing the summaries, figures and validation outputs. Do not delete or overwrite the released repository partitions.

## 4 Experimental Design, Materials and Methods

### 4.1 Scenario structure

The four simulation blocks and their scenario counts are summarized in Table 5. The core block uses sample sizes 20, 30, 50, 100 and 200. It crosses these values with three lifetime models and three censoring profiles. Each design has a null scenario, a fixed alternative and a local alternative. The extension blocks use sample sizes 50 and 100 with moderate censoring. Every null scenario has 3000 replicates. Every alternative scenario has 1500 replicates. This gives 648,000 replicate records.

**Table 5:** Simulation blocks and scenario counts.

Block	Purpose	Scenarios
Core independent	Model, sample size, censoring profile and alternative	135
Dependence	Copula, Kendall tau, model and sample size	108
Censoring comparison	Type-I, Type-II and hybrid schemes	36
Higher K	Three and five competing causes	24
Total	Complete publication design	303

### 4.2 Lifetime generation

Each subject has K latent failure times. The minimum of these latent times is the uncensored event time. The index of this minimum determines the event cause. The coefficient-allocation weights sum to one. The weights are (0.67, 0.33) for K = 2; (0.55, 0.30, 0.15) for K = 3; and (0.40, 0.25, 0.15, 0.12, 0.08) for K = 5.

For cause j, the cumulative hazard functions are defined as follows.

(1) $$\text{Exponential}:H_{j}\left(t\right)=\theta_{j}t$$

(2) $$\text{Weibull}:H_{j}\left(t\right)=\theta_{j}t_{j}^{a}$$

(3) $$\text{Gompertz}:H_{j}\left(t\right)=\theta_{j}\left[\text{exp}\left(\gamma_{j}t\right)-1\right]/\gamma_{j}$$

The Weibull shape αⱼ is fixed at 2. The Gompertz growth values γⱼ are equally spaced from 0.20 to 0.50. Shapes and growth values are treated as known in the fitted model.

The total baseline coefficient is 1.50 for the exponential model, log(2) for Weibull and 0.60 for Gompertz. The allocation weights divide that total among the causes. Inverse cumulative hazards transform uniform random values into latent times.

### 4.3 Dependence generation

The core, censoring and higher-K blocks use independent uniforms. The dependence block uses bivariate Clayton, Frank or Gumbel copulas [11,12]. Dependence is imposed on the bivariate survival uniforms Uⱼ = exp{−Hⱼ(Tⱼ)}. Latent times are obtained using Tⱼ = Hⱼ⁻¹(−log Uⱼ). Therefore, Clayton, Frank and Gumbel describe dependence between the latent survival uniforms. Kendall tau is 0.25, 0.50 or 0.75. The analysis still uses the independence likelihood. This block therefore measures sensitivity to a wrong independence assumption. Its null rejection rates are not conventional Type-I errors from a correctly specified model.

### 4.4 Censoring

Type-I censoring stops at a calibrated time T₀. Type-II censoring stops at the planned rth failure. The hybrid rule stops at the earlier of these limits. The high-, moderate- and low-censoring profiles use target failure fractions q = 0.25, 0.50 and 0.75, respectively. For Type-II and hybrid censoring, the planned failure count is *r* = max{1, floor(qn)}. For Type-I and hybrid censoring, T₀ is calibrated as the corresponding empirical quantile of the first-event time under the matching null model. Each publication calibration uses 20,000 simulated observations. The realized failure fraction is reported for every replicate and need not equal the target failure fraction.

### 4.5 Null and alternative settings

The null hypothesis concerns the coefficient for cause 1. Under the fixed alternative, this coefficient is increased by 25%. The local alternative uses an absolute model-scale shift, θ₁(true) = θ₁(null) + 1/√n. The other cause-specific coefficients do not change. Because this shift is absolute, its relative size differs across lifetime models. Cross-model comparisons of local-alternative rejection rates must therefore be interpreted cautiously.

### 4.6 Estimation and testing

Let $Y_{i}$ denote the observed follow-up time for subject i, and write the cause-specific cumulative hazard as

(4) $$H_{j}\left(t\right)=\theta_{j}G_{j}\left(t\right).$$

For cause j, the cumulative exposure is

(5) $$E_{j}=\sum_{i=1}^{n}G_{j}\left(Y_{i}\right),$$

where $G_{j}\left(t\right)=t\;$for the exponential model, $G_{j}\left(t\right)=t^{\alpha_{j}}$for the Weibull model, and

(6) $$G_{j}\left(t\right)=\frac{\text{exp}\left(\gamma_{j}t\right)-1}{\gamma_{j}}$$

for the Gompertz model. For $E_{j}>0$, the cause-specific coefficient has the closed-form estimate

(7) $${\hat{\theta }}_{j}=\frac{d_{j}}{E_{j}},$$

where $d_{j\;}$is the number of observed events attributed to cause j.

The log likelihood is

(8) $$l\left(\theta \right)\;=\;\sum_{j\;=\;1}^{K}\left[d_{j}\;log\left(\theta_{j}\right)\;-\;\theta_{j}E_{j}\right]\;+\;C,$$

where C does not depend on θ. This form avoids numerical optimizer failure.

The Wald, likelihood-ratio and score statistics for testing H₀: θ₁ = θ₁₀ are

(9) $$W\;=\;\frac{{\left({\hat{\theta }}_{1}\;-\;\theta_{10}\right)}^{2}}{\frac{{{\hat{\theta }}_{1}}^{2}}{d_{1}}},$$

(10) $$LR\;=\;2\left\{l\left(\hat{\theta }\right)\;-\;l\left(\tilde{\theta }\right)\right\},$$

and

(11) $$S\;=\;\frac{{\left(\frac{d_{1}}{\theta_{10}}\;-\;E_{1}\right)}^{2}}{\frac{E_{1}}{\theta_{10}}},$$

respectively. Here, θ̂ is the unrestricted estimate, and θ̃ is the restricted estimate obtained by setting θ₁ = θ₁₀. Because the likelihood is separable across causes, the unrestricted estimates of the nuisance parameters remain unchanged under this restriction.

Each statistic is compared with the upper tail of a chi-square distribution with one degree of freedom at α = 0.05. If cause 1 has no observed event, then d₁ = 0, and its estimate lies on the boundary. The Wald statistic and its normal interval are then unavailable. The likelihood-ratio and score statistics remain computationally available. Their availability does not imply that the asymptotic chi-square approximation is exact in sparse or boundary cases. A record with an interior target estimate and at least one zero-event nuisance cause is classified as SUCCESS_WITH_BOUNDARY_NUISANCE.

### 4.7 Monte Carlo uncertainty

Throughout this article, each rejection proportion is calculated using the test-specific available denominator. The Wald rejection proportion is therefore conditional on availability when cause 1 has at least one observed event. The release reports the rejection count, rejection proportion, conventional Monte Carlo standard error, Jeffreys posterior mean, Jeffreys posterior standard deviation and 95% Wilson interval [9,13,14]. It also reports lower and upper failure bounds based on all requested replicates. These bounds show the possible range when unavailable replicates are treated as either non-rejections or rejections. These fields make zero-event and unavailable-test handling explicit. Cause-specific bias is the mean estimate minus the true coefficient, and RMSE is the square root of the mean squared estimation error. These quantities are reported by scenario and are not pooled across models with different coefficient scales.

### 4.8 Bootstrap validation

Parametric bootstrap validation uses one independent, moderate, hybrid-censored null condition for each lifetime model at n = 50. Thirty successful parent datasets are used per model. For each parent dataset, 499 new datasets are generated from the fitted cause-specific parameters. The Type-I stopping time $T_{0}\;$and the planned failure count rare retained from the parent scenario and are not recalibrated using the fitted bootstrap parameters. Only bootstrap datasets with an available cause-1 estimate are retained. A parent interval is calculated only when at least 399 of the 499 bootstrap estimates are successful. A percentile interval is calculated from the successful bootstrap estimates of the cause-1 coefficient [15]. This is a limited validation check rather than a separate simulation block.

### 4.9 Reproducibility and resume safety

Each scenario identifier uses the first 16 hexadecimal characters of the SHA-256 digest of its canonical scenario definition. Each replicate seed is a deterministic hash of the master seed, pipeline version, scenario identifier and replicate number. The master seed is 20,260,817. Partitions are written atomically. Before reuse, the notebook checks their schema, pipeline version, identifiers and complete replicate sequence. The Colab workflow uses two CPU workers and thread-based scenario jobs; a GPU is not required.

The saved publication execution reported RESUME_VALIDATION because all 303 released partitions were already present. Before reuse, every partition passed checks of its schema, pipeline version, scenario identifier, replicate identifiers and complete replicate sequence. Consequently, no partition was regenerated during this final validation execution. The RESUME_VALIDATION status therefore represents verified reuse of the complete release rather than a partial or interrupted run.

Evidence from the original fresh-generation execution is retained in data/generation_runtime_report.csv, which records the original non-zero generation timings. The file data/generation_current_run.csv records the status and timing of the final resume-validation execution. To perform an independent fresh run, users should direct the notebook to a new empty output directory and execute all cells in their original order without changing the master seed or simulation settings. When no valid partitions are present, the notebook generates all 303 scenario partitions before rebuilding the subject-level sample, summaries, figures and validation outputs. The released partitions do not need to be deleted. The executed environment used Python 3.13 and is recorded with the release metadata.

### 4.10 Release-level reconciliation

The release-level validation recalculated every scenario summary directly from its Parquet partition. It checked all 648,000 records, test-specific available denominators, rejection counts, rejection proportions and the two boundary classifications. The recalculated values were compared with data/summary_size_power.csv for all 303 scenarios.

The representative subject-level file was checked as 465 scenario-replicate groups. For each group, the validation compared the subject count, seed, stopping rule, latent minimum, event cause, cause-specific counts, exposures and coefficient estimate with the matching replicate partition. A separate calculation recomputed the coefficient estimates, Wald, likelihood-ratio and score statistics, information terms and p-values for 27 representative records using a numerical tolerance of 1e-12.

## Limitations

The dataset consists entirely of computer-generated observations and does not represent patients, machines or an observed population. Its coverage is limited to exponential, Weibull and Gompertz lifetime models, two, three or five competing causes, and sample sizes between 20 and 200. The scenarios contain no covariates or missing cause labels. Weibull shape and Gompertz growth parameters are fixed within the specified designs and treated as known.

Dependent latent times are included only for two-cause scenarios using Clayton, Frank and Gumbel copulas at three specified Kendall’s tau values. Other dependence structures and dependence among three or more causes are not included. The censoring designs are restricted to the documented Type-I, Type-II and hybrid schemes.

The subject-level file contains 35,250 rows from five replicates for each of 93 selected moderate-censoring null conditions. It therefore represents only a subset of the simulated studies; complete subject-level observations for all 648,000 replicates are not stored in the release. The bootstrap component contains 30 parent datasets per lifetime model and covers only the selected validation conditions.

## Ethics Statement

The authors have read and follow the ethical requirements for publication in Data in Brief. This work used only computer-generated synthetic data and did not involve human subjects, animal experiments or data collected from social media platforms. No patient records or identifiable personal information were used. Ethics approval and informed consent were not required.

## CRediT Author Statement

**Sabrigiriraj M:** Conceptualization, Methodology, Visualization, Data curation, Supervision; **Manoharan K:** Conceptualization, Methodology, Supervision; **Senthil Madasamy N:** Conceptualization, Methodology, Writing – original draft; **Govindaraj V:** Data curation, Writing – original draft; **Prathipa K:** Data curation, Writing – review & editing; **Ravi T:** Data curation, Writing – review & editing.

## Declaration of Generative AI and Ai-Assisted Technologies

During the preparation of this work, the authors used ChatGPT by OpenAI to assist with code debugging, manuscript organization and language editing. After using this tool, the authors reviewed and edited the code and text, executed the complete notebook, checked the generated files and numerical results, and take full responsibility for the content of the publication.
